# Validation of the Scale of Basic Psychological Needs towards Physical Exercise, with the Inclusion of Novelty

**DOI:** 10.3390/ijerph17020619

**Published:** 2020-01-18

**Authors:** Ruben Trigueros, Joaquín F. Álvarez, Adolfo J. Cangas, José M. Aguilar-Parra, Cristina Méndez-Aguado, Patricia Rocamora, Remedios López-Liria

**Affiliations:** 1 Department of Psychology, Hum-878 Research Team, Health Research Centre, University of Almería, 04120 Almería, Spain; jalvarez@ual.es (J.F.Á.); cristinamendezaguado@gmail.com (C.M.-A.); 2 Department of Psychology, Hum-760 Research Team, Health Research Centre, University of Almería, 04120 Almería, Spain; ajcangas@ual.es; 3 Department of Nursing Science, Physiotherapy and Medicine, Hum-498 Research Team, Health Research Centre, University of Almería, 04120 Almería, Spain; rocamora@ual.es (P.R.); rll040@ual.es (R.L.-L.)

**Keywords:** self-determination theory, intrinsic motivation, basic psychological needs, physical activity

## Abstract

The purpose of this study was to validate and adapt to the Spanish context of Physical Education, the Spanish version of the Scale of Basic Psychological Needs in the context of physical exercise, with the incorporation of novelty to the scale. The sample that took part in the study was 2372 people from 16 to 48 years old from the province of Almeria. In order to analyze the psychometric properties of the scale, several analyses have been carried out. The results have offered support both for the eight-factor structure and for the higher-order double model where the eight subscales are joined into two constructs called frustration and satisfaction. The structure of both models was invariant with respect to gender and age. Cronbach’s alpha values were above 0.70 in the subscales and scales; and adequate levels of temporal stability. In addition, the subfactors pertaining to the satisfaction of basic psychological needs positively predicted the intrinsic motivation for physical activity, while each of the subfactors of the frustration of psychological needs predicted it negatively. The results of this study provide evidence of the reliability and validity of the BPNS in the Spanish context of physical activity.

## 1. Introduction

In recent years, various studies in the field of physical activity and sport have shown the multiple benefits at the physical, cognitive, psychological, emotional, and social levels that are linked to the regular practice of physical activity [1,2]. In this sense, the regular practice of physical activity is linked to the reduction of heart problems, reduction of hypertension, stress, depression, and feelings of loneliness [3]. Despite these benefits, regular physical activity is still very low, with around 61% of the world’s population failing to reach the 300 min per week recommended by the World Health Organization [4]. For this reason, several studies have suggested that experiences linked to the practice of physical activity should be positive in order to encourage adherence [5]. Thus, it is especially important to understand both the negative and positive experiences people have had while practicing PA [6,7]. This study aims to adapt and validate to the Spanish context a scale with which to evaluate basic psychological needs in the area of physical exercise, incorporating novelty.

From the Self-Determination Theory (SDT; [8]) it is suggested the existence of a series of psychological needs that are basic and present in all human beings. These needs are necessary for the psychological and social well-being and health of all people, promoting personal growth and development [9]. These basic psychological needs are represented by three essential factors: autonomy, which refers to the fact that people are agents of their own action without external impositions; competence, which refers to having a sense of ability and effectiveness in the action being carried out; and relationship, which refers to the feeling of being integrated into a social group of relevance to the individual [9,10]. However, at present several studies have justified and proposed the incorporation of a fourth basic psychological need called novelty [11,12]. This psychological need refers to the search for new experiences and sensations that have not been experienced or lived before and therefore deviates from the daily routine [13]. This need had previously been mentioned by Deci and Ryan [8] when they defined intrinsic motivation as the active commitment that people acquire with the actions that they find interesting and new and that represent an optimal challenge. This new psychological need is identified with exploration and spontaneous interest, assimilation and improvement as a form of cognitive, emotional and social development [8].

The four basic psychological needs are interrelated, so that if one of them diminishes the others will also diminish [13,14]. Therefore, people who feel influenced when they make decisions, unable when they practice physical activity, excluded from the social reference group, and find the activities monotonous and repetitive, would experience a frustration of their basic psychological needs, which are related to the abandonment of activity, lack of commitment, deficits in interpersonal relationships, and, in short, the manifestation of maladaptive behaviors [15]. Conversely, if people feel autonomous when they make decisions, feel competent when they carry out their activities, accepted and integrated into their social reference group, and the activities turn out to be different and attractive, they will experience a satisfaction of psychological needs, which is related to the learning of new skills, commitment to learning, improvement of interpersonal relations and the manifestation of adaptive behaviours [16].

At present, there are many studies from SDT, especially from the field of Physical Education (PE) classes, which have analyzed the relationship between novelty and the autonomous motivation of students. In this sense, a study carried out by Trigueros, et al. [17] has shown how those students in PE who had high levels of satisfaction of novelty also had high levels of autonomous motivation for PE classes showing that both factors are related. Thus, it is necessary for teachers to promote new and continuous experiences during the PE classes so that their students feel attracted and interested in order to increase the motivation of the students. From the field of physical activity, we have no record of studies that have considered this factor. However, there are some studies from the field of physical activity that have linked SDT to factors with similar characteristics to those described by the novelty such as: the search for new sensations [18], curiosity [19] and desire [20]. Although it is true that the approach that researchers have used of these variables is close to what has been defined as novelty, the focus of these investigations is different in terms of a possible establishment of a new basic psychological need (for a greater understanding, see González-Cutre, et al., [11]).

Despite this dual validation of basic psychological needs, studies on them are highly fragmented. To such an extent that the study of the inexistence of a questionnaire that takes into consideration both aspects (see, [13,14,21,22]). In this sense, a study centred on psychological needs towards life and carried out by Longo, Alcaraz-Ibáñez and Sicilia [23] with university students showed the importance of uniting both valences in order to have a general vision of the influence of psychological needs and their effects.

Based on this background, the objective that we propose is to adapt and validate the Basic Psychological Needs Scale (BNPS) to the Spanish context of physical exercise, incorporating the fourth novelty need. Joining in the same scale the satisfaction and frustration of the psychological needs in order to measure both the positive and negative side. The CFA of the proposed instrument is expected to provide adequate adjustment rates for the eight-factor correlated model and the higher-order model. Both models are expected to be invariant with respect to gender and age. In addition, the internal consistency of the factors and their temporal stability is expected to be adequate. A structural equation model will be made to show evidence of validity of the BPNS criteria by analyzing the predictive relationships of each of the psychological needs with respect to the intrinsic motivation for physical activity.

## 2. Method

### 2.1. Participants

A total of 2372 people between the ages of 16 and 48 (*M* = 28.39; *SD* = 12.30) taking part in physical activity in various sports centres in Andalusia.

In addition, a different sample from the first one participated in the present study in order to carry out the temporal stability, which was made up of 871 persons between 17 and 51 years old (*M* = 27.65; *SD* = 9.68). This second group completed the scale on two occasions, with a time interval of two weeks between both data collection.

A third independent sample of 2138 people aged 16–49 was used to analyse the predictability of the scale through a structural equation model.

The sample used was non-probabilistic incidental, depending on those sports centres and people who had access to them.

### 2.2. Instruments

#### 2.2.1. Basic Psychological Needs Scale (BPNS) towards Physical Activity

Satisfaction of basic psychological needs. The validated and adapted version of the Satisfaction of Basic Psychological Needs Scale was used in the exercise by Sánchez and Nuñez [21] whose factors are autonomy, competence, and relationship. The final scale comprises a total of 12 items distributed among the three factors that make up the scale: autonomy, competence, and relationship. In order to measure the satisfaction of novelty towards physical exercise, the items corresponding to the factor with the same name belonging to the scale of Satisfaction of Psychological Needs towards Physical Education classes of Trigueros, et al., [13] were adapted. This factor is made up of six items.

Frustration of psychological needs. The adapted version of the Psychological Needs Frustration Scale was used in physical exercise (PNFS; [22]). The scale was preceded by the heading “In my PE classes...” and consists of 12 items, distributed equally among each of the factors that make up the scale (i.e., autonomy, competence, relationship with others). In order to measure the frustration of novelty, the items corresponding to the factor with the same name belonging to the scale of Frustration of Psychological Needs were adapted to the Physical Education classes of Trigueros, et al., [14]. This factor is made up of five items.

Each of the above scales is of the Likert type, ranging from 1 (not true at all) to 7 (totally true).

#### 2.2.2. Intrinsic Motivation

The factor with the same name from the Behaviour Regulation in Practice Questionnaire (BREQ-3) by González-Cutre, Sicilia and Alberto Fernández was used [24]. The scale was headed by the following heading, “I do physical exercise...” and the intrinsic motivation factor consisted of 4 items. The questionnaire is of the Likert type, ranging from 0 (nothing true) to 4 (totally true).

### 2.3. Procedure

Some sports centres were asked to collaborate in the research, so that they could allow us to reach the people who come to the sports centre to do physical exercise and explain to them the objective of the study. In order to participate, it was necessary to fill out the informed consent form. When filling in the questionnaires, we insisted on the anonymity of the answers, emphasizing that they were being asked for their own opinions and that participation was voluntary. A member of the research group was present while people completed the questionnaires to answer any questions they might have. All ethical procedures for data collection were respected, taking about 20 min to complete the questionnaires.

### 2.4. Data Analysis

To analyze the psychometric properties of the BNPS towards physical exercise, a series of analyses were carried out in order to be able to determine its validity and reliability. Firstly, two confirmatory factor analyses (CFAs) were carried out in order to test the factor structure of the questionnaire. Then, a multi-group analysis was performed in relation to sex and age in order to determine if the questionnaire is understood in a similar way without age or sex being determinants. Subsequently, the statistical-descriptive analysis and the internal consistency analysis were carried out using Cronbach’s alpha in order to test the reliability of the instrument and a temporal stability analysis using a test-retest. Finally, a criteria analysis was carried out through a structural equation model, where each of the factors that make up the scale was related to the intrinsic motivation. The statistical packages SPSS 25.0 and AMOS 22.0 (IBM, Armonk, NY, USA) were used for the data analysis.

The maximum likelihood estimation method was used along with the bootstrapping procedure for the AFC and the path analysis. In order to accept or reject the model tested, a set of adjustment indexes was taken into consideration [25]: Since χ^2^ is very sensitive to sample size, χ^2^/df was used and values below 3 were considered acceptable; IFC (Comparative Fit Index) and IFI (Incremental Fit Index) show a good fit with values equal to or higher 0.95; RMSEA (Root Mean Square Error of Approximation) plus its confidence interval (CI) at 90%, and SRMR (Standardized Root Mean Square Residual) are considered acceptable with values equal to or less than 0.06 and 0.08, respectively.

## 3. Results

### 3.1. Confirmatory Factor Analysis

**Figure 1:**
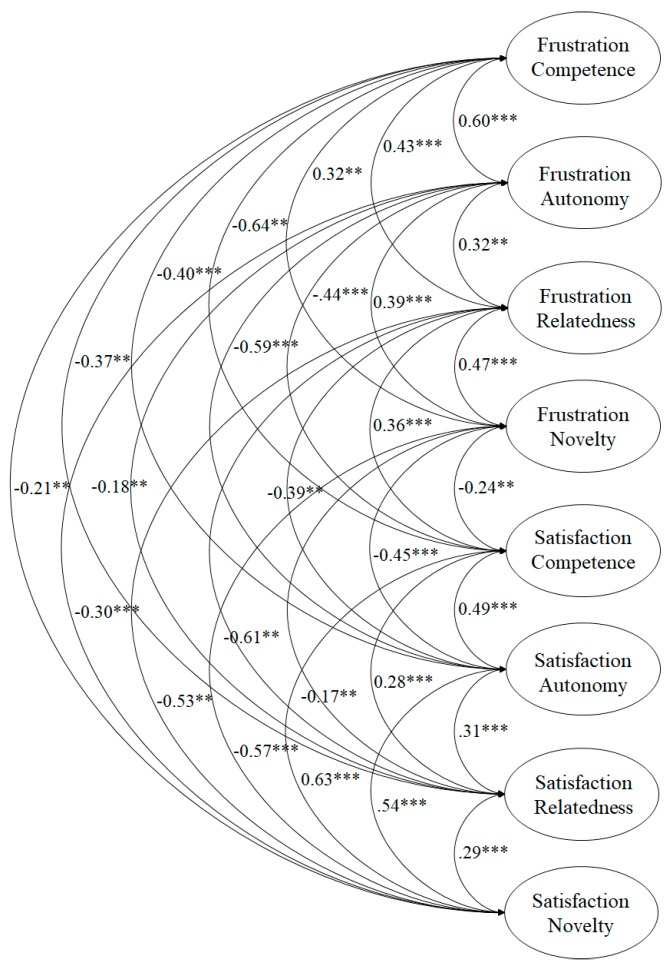
Confirmatory factor analysis of the Basic Psychological Needs Scale (BPNS).

The fit indices of the model tested (Figure 1) revealed the following settings: χ^2^ (532. N = 2372) = 1044.73, *p* < 0.001; χ^2^/df = 1.96; CFI = 0.95; IFI = 0.95; RMSEA = 0.052 (CI 90% = 0.046–0.057); SRMR = 0.032. Standardized regression weights ranged from 0.70 to 0.86 and were statistically significant (*p* < 0.001).

**Figure 2:**
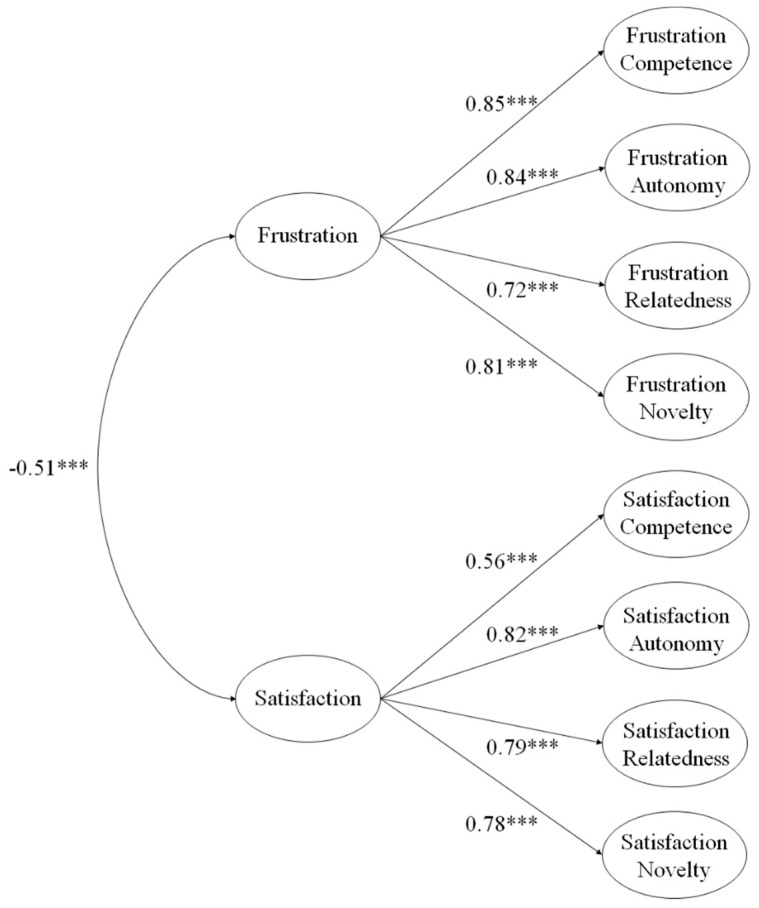
Higher order confirmatory factor analysis of the BPNS.

Once the model was determined, a higher order model was tested (e.g., the eight first order factors converging into two higher order factors called frustration and satisfaction). The adjustment indices of this model are as follows (Figure 2): χ^2^ (551. *N* = 2372) = 958.04, *p* < 0.001; χ^2^/df = 1.74; CFI = 0.96; IFI = 0.96; RMSEA = 0.052 (CI 90% = 0.047–0.058); SRMR = 0.041. All standardized regression weights were significant (*p* < 0.001), with 0.85 for frustration of competence, 0.84 for frustration of autonomy, 0.72 for frustration of relatedness, 0.81 for frustration of novelty, 0.56 for satisfaction of competence, 0.82 for satisfaction of autonomy, 0.79 for satisfaction of relatedness, and 0.78 for satisfaction of novelty. As for the correlations between the higher order factors, they were −0.51, being statistically significant (*p* < 0.001).

#### Gender and Age Invariance Analysis

A multi-group analysis was carried out in order to find out whether the factor structure of the model is invariant with respect to gender and age. As shown in Table 1 and Table 2 for the eight-factor model, no significant differences were observed in the statistic χ2 between model 1 (non-constrained model) and model 2 (model with invariant measurement weights) and yes with respect to model 3 (invariant structural covariance models) and model 4 (invariant residual measures model). In addition, Table 1 and Table 2 show the adjustment rates for the six models compared within the higher-order two-factor structure. Likewise, no significant differences were found between model 1 and model 2 and whether there were significant differences between model 3, model 4 (structural covariance model), model 5 (structural invariant residuals model), and model 6 (invariant residual measures model). The differences between models 1 and 2 constitute a minimum criterion to be able to say that the factor structure of the questionnaire, a four-factor model and the higher order model, is invariant with respect to gender [26].

### 3.2. Descriptive Statistics, Correlation and Reliability Analysis

In Table 3, the means, standard deviation and bivariate correlations are shown. The correlations reflected a positive association between those factors linked to each other and a negative association between the opposites. In addition, Table 3 shows that the internal consistency analysis reflected Cronbach’s alpha values greater than 0.70 [27,28] for each of the factors.

In the temporal stability analysis, intra-class correlation coefficients (ICC) and their confidence intervals (CI) were calculated.

### 3.3. Criteria Validity Analysis 

**Figure 3:**
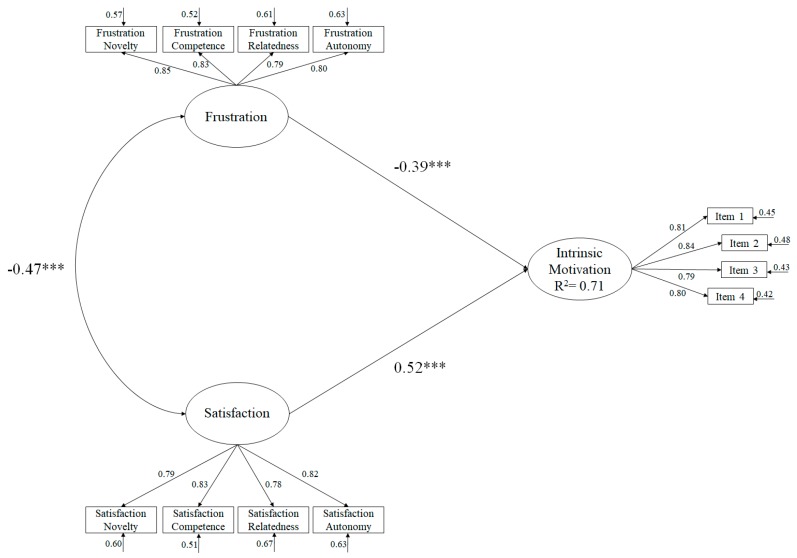
Structural equation model.

In order to analyze the criterion validity of the BPNS, a structural equation model was made to analyze the predictability of the scale. The fit indices of the model tested (Figure 3) revealed the following fit indices: *χ^2^* (51. *N* = 2138) = 147.67, *p* < 0.001; *χ^2^/df* = 2.89; IFC = 0.96; IFI = 0.96; RMSEA = 0.051 (CI 90% = 0.049–0.061); SRMR = 0.038.

## 4. Discussion

The present study aims to determine the validity of the factor structure, internal consistency, temporal stability and predictive validity of the BPNS towards physical exercise. The results have shown that the BPNS as an instrument with adequate validity and reliability to evaluate the positive and negative aspects of basic psychological needs towards physical exercise, also incorporating novelty. In this way, an effective tool is available that can help researchers and professionals to understand in greater depth the predictive effects of basic psychological needs on the adaptive and maladaptive behaviors of people who engage in any type of physical activity [29,30].

The first CFA revealed that the factorial structure of the BPNS showed adequate adjustment rates for the eight-factor model, showing a positive relationship between those needs with the same root and a negative relationship between those needs with different roots. These results are similar to various studies that have been carried out to date [10,11,12,14], and are in line with the postulates of the SDT [5,8] where the reciprocity between each of the psychological needs is defended, also incorporating the novelty, continuing the path of previous studies (e.g., González-Cutre et al., [11]; Kashdan, and Silvia, [31]). As for the second CFA, the factor structure of the higher-order dual model revealed acceptable adjustment rates, showing a negative correlation between frustration and satisfaction. This model is interesting because it supports the use of an overall value composed of the mean of the sub-factors, which can be used by researchers to simplify models where several constructs are present. Furthermore, its use is justified since a study by Gagné, Ryan, and Bargmann [32] suggested that needs tend to function as a single “body” in different situations.

On the other hand, the reliability and temporal stability analyses revealed acceptable adjustment rates for all eight subscales and higher order factors. As for the mutli-group analysis, it showed that the structure of the eight-factor model and the higher-order model of the BPNS were invariant with respect to gender and age. These results support the use of the questionnaire in future research where it is intended to compare means between boys and girls as well as between different ages.

Finally, evidence of predictive validity for the scale was found through the structural equation model. This analysis showed that each of the factors pertaining to the satisfaction of basic psychological needs positively predicted the intrinsic motivation, while the frustration of basic psychological needs predicted it negatively. These results are similar to previous studies where satisfaction of psychological needs was positively related to intrinsic motivation [33,34] and frustration of psychological needs was negatively related to intrinsic motivation [35,36]. These relationships appear to support the postulates of SDT that the frustration of psychological needs may lead to the search for a collateral satisfaction that compensates for this feeling of frustration by producing a series of disadaptive consequences or inhibition that may be contrary to personal well-being, and conversely, the satisfaction of basic psychological needs is closer to adaptive behaviors and continued participation that promote personal well-being [37].

Despite the results achieved by this study, a number of limitations should be highlighted. In this sense, future studies should continue to analyze the factorial structure of the questionnaire since the creation and/or adaptation of a questionnaire is a continuous process that requires an in-depth analysis of its functioning in different populations with equally different sociodemographic characteristics.

## 5. Conclusions

BPNS towards physical this scale exercise offers researchers a valid and effective tool that can help measure people’s perceived basic psychological needs towards physical exercise. Moreover, it is in line with the postulates of the SDT and will allow further assessment of the contextual factors inherent to physical activity can undermine or promote psychological well-being by promoting personal development.

## Figures and Tables

**Table 1:** Multi-group Gender Invariance Analysis.

**Eight-Factor Primary Order Model**									
**Models**	***χ^2^***	***df***	***χ^2^/df***	**Δ*χ^2^***	**Δ*df***	**CFI**	**IFI**	**SRMR**	**RMSEA (CI 90%)**
Model 1	1668.77	1158	1.56	-	-	0.95	0.95	0.037	0.046 (0.042–0.050)
Model 2	1715.52	1160	1.57	46.75	27	0.95	0.95	0.037	0.046 (0.042–0.050)
Model 3	1768.97	1162	1.57	100.20 **	63	0.94	0.94	0.037	0.046 (0.042–0.050)
Model 4	1847.72	1166	1.59	178.95 ***	98	0.94	0.94	0.039	0.047 (0.043–0.051)
**Higher-Order Two-Factor Model**									
**Models**	***χ^2^***	***df***	***χ^2^/df***	**Δ*χ^2^***	**Δ*df***	**CFI**	**IFI**	**SRMR**	**RMSEA (CI 90%)**
Model 1	1720.87	1102	1.56	-	-	0.95	0.95	0.035	0.046 (0.041–0.050)
Model 2	1769.50	1129	1.57	48.62	27	0.95	0.95	0.035	0.046 (0.041–0.050)
Model 3	1772.28	1135	1.56	51.41 *	33	0.95	0.95	0.035	0.046 (0.041–0.050)
Model 4	1773.28	1138	1.56	52.41 *	36	0.95	0.95	0.036	0.046 (0.041–0.050)
Model 5	1801.24	1146	1.57	80.37 **	44	0.95	0.95	0.035	0.046 (0.041–0.050)
Model 6	1887.46	1181	1.60	166.59 ***	79	0.94	0.94	0.037	0.046 (0.043–0.051)

Note: Comparative Fit Index (CFI); Incremental Fit Index (IFI); Root Mean Square Error of Approximation (RMSEA); Standardized Root Mean Square Residual (SRMR); Confidence Interval CI; * *p* < 0.05; ** *p* < 0.01; *** *p* < 0.001.

**Table 2:** Multi-group Age Invariance Analysis.

**Modelo de Ocho Factores de Orden Primario**									
**Models**	***χ^2^***	***df***	***χ^2^/df***	**Δ*χ^2^***	**Δ*df***	**CFI**	**IFI**	**SRMR**	**RMSEA (CI 90%)**
Model 1	1831.39	1064	1.72	-	-	0.95	0.95	0.041	0.065 (0.060–0.070)
Model 2	1858.07	1091	1.70	26.70	27	0.95	0.95	0.043	0.064 (0.059–0.069)
Model 3	1945.13	1127	1.73	113.75 **	63	0.95	0.95	0.044	0.064 (0.060–0.070)
Model 4	2001.50	1162	1.72	170.12 ***	98	0.94	0.94	0.047	0.065 (0.060–0.070)
**Higher-Order Two-Factor Model**									
**Models**	***χ^2^***	***df***	***χ^2^/df***	**Δ*χ^2^***	**Δ*df***	**CFI**	**IFI**	**SRMR**	**RMSEA (CI 90%)**
Model 1	1914.13	1102	1.74	-	-	0.95	0.95	0.045	0.066 (0.060–0.071)
Model 2	1942.19	1129	1.72	28.06	27	0.95	0.95	0.045	0.065 (0.060–0.070)
Model 3	1945.80	1135	1.71	31.68	33	0.95	0.95	0.044	0.065 (0.060–0.069)
Model 4	1955.25	1138	1.72	41.12	36	0.95	0.95	0.044	0.065 (0.060–0.070)
Model 5	1989.48	1146	1.74	75.35 **	44	0.94	0.94	0.044	0.066 (0.061–0.070)
Model 6	2045.43	1181	1.73	131.30 ***	79	0.94	0.94	0.044	0.065 (0.061–0.070)

Note: ** *p* < 0.01; *** *p* < 0.001.

**Table 3:** Descriptive Statistics and Correlations between all BPNS Factors.

Factors	*M*	*SD*	α	1	2	3	4	5	6	7	8	9	10	ICC
1. Frustration of autonomy	1.88	1.10	0.87		0.62 ***	0.73 ***	0.43 ***	0.82 ***	−0.45 **	−0.25 ***	−0.41 **	−0.12 **	−0.40 **	0.77 (IC = 0.71–0.86)
2. Frustration of competence	2.17	1.14	0.86			0.81 ***	0.42 ***	0.84 ***	−0.20 **	−0.12 *	−0.15 *	−0.32 **	−0.74 **	0.83 (IC = 0.78–0.85)
3. Frustration relatedness	1.89	1.12	0.85				0.40 ***	0.83 ***	−0.34 ***	−0.37 ***	−0.23 *	−0.54 **	−0.48 **	0.81 (IC = 0.78–0.83)
4. Frustration novelty	2.00	0.95	0.82					0.80 ***	−0.36 ***	−0.56 ***	−0.41 ***	−0.19 *	−0.45 **	0.80 (IC = 0.77–0.83)
5. Frustration	1.99	1.12	0.88						−0.37 **	−0.32 **	−0.84 **	−0.13 ***	−0.73 ***	0.85 (IC = 0.69–0.85)
6. Satisfaction Autonomy	5.23	0.86	0.82							0.63 ***	0.51 ***	0.68 ***	0.85 ***	0.75 (IC = 0.71–0.83)
7. atisfaction of the competence	5.31	0.91	0.81								0.56 ***	0.62 ***	0.83 ***	0.80 (IC = 0.79–0.84)
8. Satisfaction relatedness	5.40	0.67	0.83									0.48 ***	0.81 ***	0.83 (IC = 0.79–0.86)
9. Satisfaction novelty	5.11	0.87	0.85										0.80 ***	0.86 (IC = 0.81–0.88)
10. Satisfaction	5.26	1.03	0.88											0.89 (IC = 0.81–0.90)

Note: * *p* < 0.05; ** *p* < 0.01; *** *p* < 0.001.
